# Acute and Chronic Effects of Protein Kinase-D Signaling on Cardiac Energy Metabolism

**DOI:** 10.3389/fcvm.2018.00065

**Published:** 2018-06-07

**Authors:** Ozlenen Simsek Papur, Aomin Sun, Jan F. C. Glatz, Joost J. F. P. Luiken, Miranda Nabben

**Affiliations:** ^1^Department of Genetics & Cell Biology, Faculty of Health, Medicine and Life Sciences, Maastricht University, Maastricht, Netherlands; ^2^Department of Molecular Medicine, Institute of Health Science, Dokuz Eylul University, Izmir, Turkey

**Keywords:** protein kinase D, PKD isoforms, glucose uptake, fatty acid uptake, cardiac hypertrophy, membrane substrate transporter

## Abstract

Protein kinase-D (PKD) is increasingly recognized as a key regulatory signaling hub in cardiac glucose uptake and also a major player in the development of hypertrophy. Glucose is one of the predominant energy substrates for the heart to support contraction. However, a cardiac substrate switch toward glucose over-usage is associated with the development of cardiac hypertrophy. Hence, regulation of PKD activity must be strictly coordinated. This review provides mechanistic insights into the acute and chronic regulatory functions of PKD signaling in the healthy and hypertrophied heart. First an overview of the activation pathways of PKD1, the most abundant isoform in the heart, is provided. Then the various regulatory roles of the PKD isoforms in the heart in relation to cardiac glucose and fatty acid metabolism, contraction, morphology, function, and the development of cardiac hypertrophy are described. Finally, these findings are integrated and the possibility of targeting this kinase as a novel strategy to combat cardiac diseases is discussed.

## Introduction

The heart has a high demand for energy substrates in order to sustain perpetual cycles of contraction and relaxation. The predominant substrates used under physiological conditions are (long chain) fatty acids and glucose (1). As a result, there is an almost continuous delivery of these substrates to the heart. Substrate uptake by cardiac myocytes occurs by facilitated diffusion and involves specific membrane transporters, i.e., CD36 (SR-B2) for fatty acids (2) and members of the GLUT (SLC2) family of glucose transporters for glucose (3). Importantly, for both substrates, the transporter-mediated uptake presents the rate-controlling site in their cellular utilization (4, 5). The heart possesses mainly two types of glucose transporters, i.e., GLUT1 and GLUT4. GLUT1 is known as the constitutive glucose transporter that permanently resides at the sarcolemma. In contrast, GLUT4 is present mainly in intracellular compartments from where it can be induced to translocate to the cell surface. Hence, GLUT1 mainly contributes to basal (non-stimulated) glucose uptake and GLUT4 to glucose uptake induced by a variety of physiological and pharmacological stimuli (3, 6). Because GLUT4 is considerably more abundant in the heart than GLUT1 (7), the inducible component of glucose uptake is critically important in cardiac energy metabolism. Increased cardiac contractile activity and increased circulating insulin levels are the main physiological stimuli for inducing GLUT4 translocation to the sarcolemma and increasing cellular glucose uptake. In both cases the cellular signaling pathways involved have been largely disclosed. For regulation of contraction-induced GLUT4 translocation, the key signaling kinase was long thought to be AMP-activated kinase (AMPK) (8). However, more recently, work in our laboratory has revealed that cardiac contraction-induced GLUT4 translocation requires the additional activation of another kinase, namely protein kinase D1 (PKD1) (9, 10). Conversely, PKD1 appears not involved in insulin-stimulated glucose uptake (9, 10).

The signaling kinase PKD1 is involved in several receptor-mediated signal transduction cascades that function in multiple fundamental cellular processes, such as cell proliferation and differentiation, membrane trafficking, immune response, inflammation, angiogenesis, and cancer [for review see 11, 12). With respect to the cardiovascular system, PKD1 has been studied mainly in the context of cardiac hypertrophy and remodeling (13). The notion that PKD1 also functions in the contraction-induced regulation of cardiac glucose uptake indicates that PKD1 signaling is involved in both acute and chronic adaptations of the heart to external stimuli. Interestingly, PKD1's closely related relatives PKD2 and PKD3 have also been implicated in the regulation of cardiac glucose uptake and cardiac hypertrophy (12), together resulting in a complex signaling network.

The aim of this review is to provide mechanistic insight into the role of PKD—with focus on PKD1—in the healthy heart as well as in the development of cardiac disease. For this, we will first provide an overview of the PKD family, mostly focused on PKD1 activation, then we will discuss the various roles of the PKDs in the heart related to cardiac glucose and fatty acid metabolism, contraction, morphology and function. Finally, we will integrate these findings and discuss the application of PKD1 as target for therapeutic intervention.

## PKD1 and different routes to activation

The protein kinase-D family makes up a family of three closely related Ser/Thr kinases: PKD1, PKD2, and PKD3. They were formerly categorized within the protein kinase C family based on the presence of two regulatory C1 domains, which are homologous to the diacylglycerol (DAG) binding domain of the PKC members. Hence, PKD1 is also referred to as PKCμ, and PKD3 as PKCν. Yet, the PKDs display unique structural properties that set them apart from the PKCs. Namely, they lack an auto-inhibitory pseudo-substrate region, a characteristic feature of the PKCs, but additionally possess a pleckstrin homology (PH) domain, which is absent in the PKCs. Moreover, the catalytic domain is different from the PKCs and shows more homology with that of the calcium/calmodulin-dependent kinases (CaMKs).

**Figure 1:**
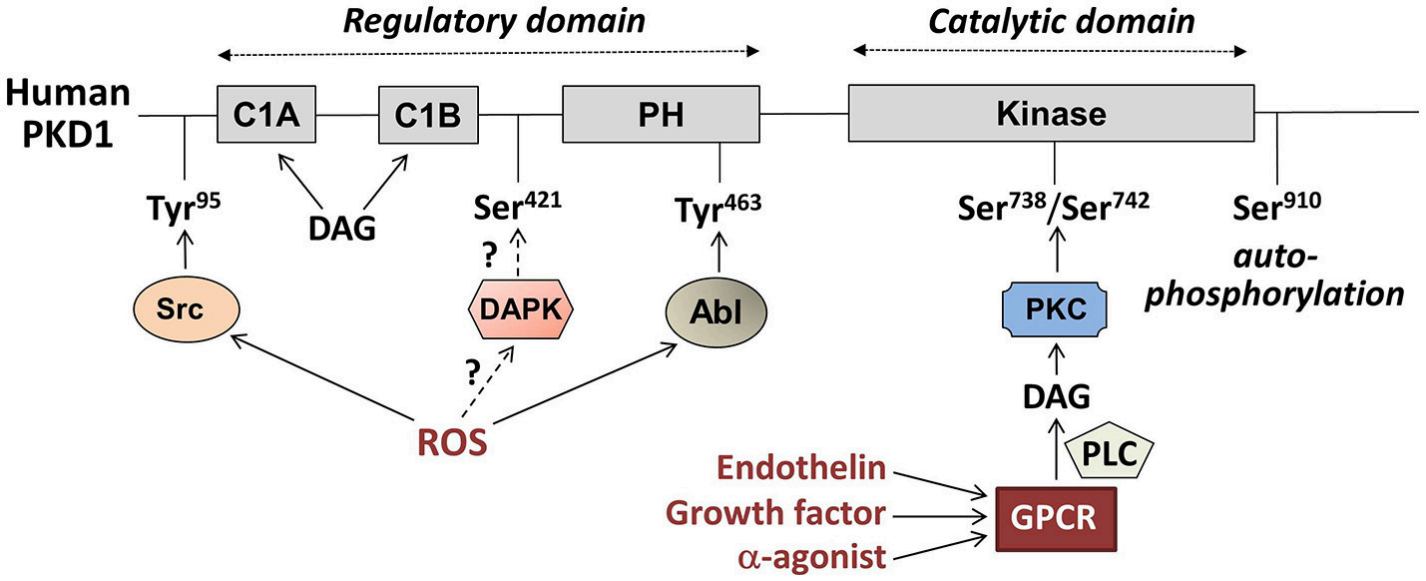
Main regulatory phosphorylation sites of human Protein Kinase D1 (PKD1) and its upstream pathways of activation. PKD1 consist of two main domains: a regulatory domain **(Left)** and a catalytic domain **(Right)**. The locations of the regulatory tyrosine (Tyr) and serine (Ser) phosphorylation sites are indicated. DAG, diacylglycerol; PH, pleckstrin homology domain; DAPK, death-associated protein kinase; ROS, reactive oxygen species; PKC, protein kinase C; PLC, phospholipase C; GPCR, G protein-coupled receptor.

**Figure 2:**
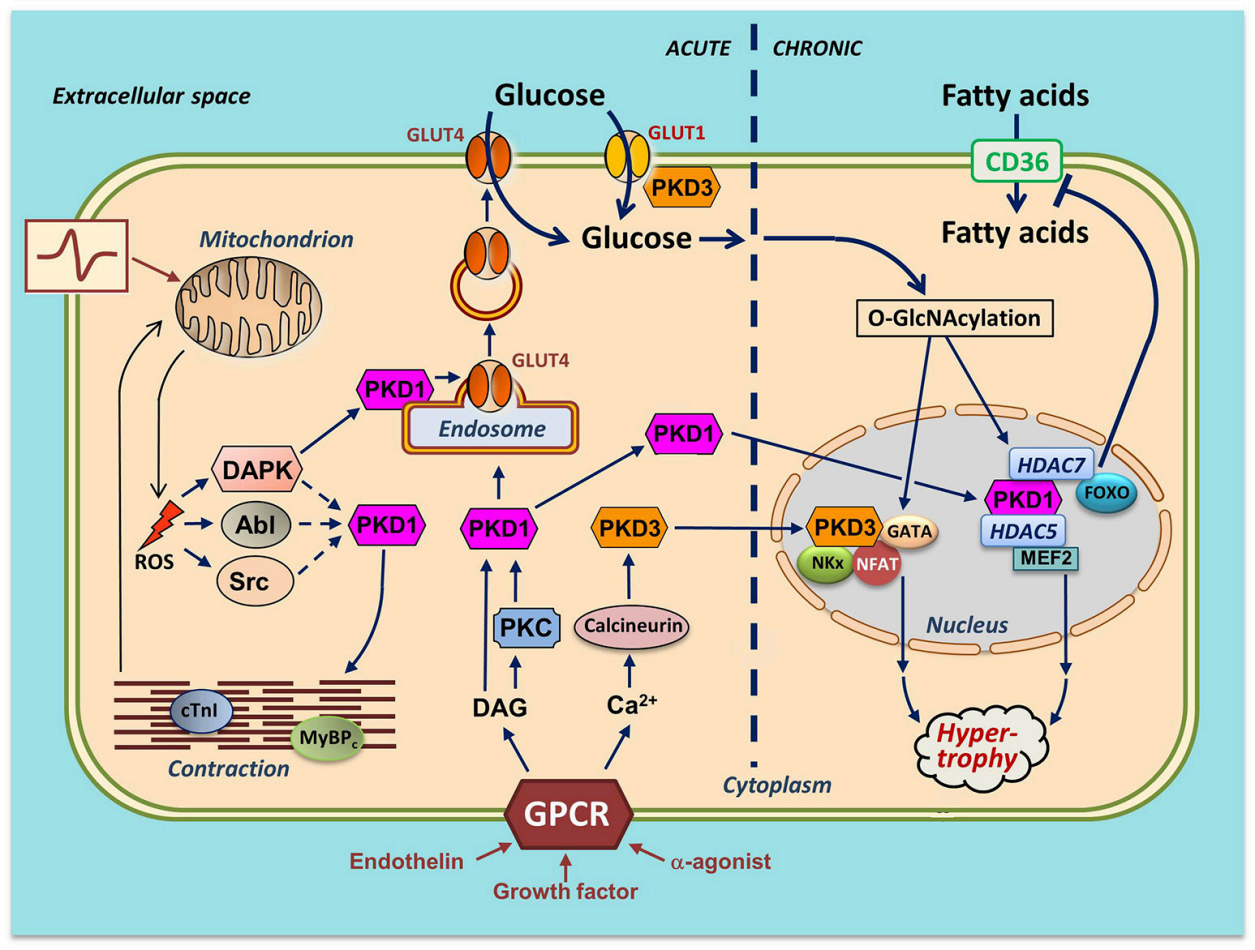
Integration of actions of PKD1 and PKD3 in cardiac energy metabolism—a hypothetical scheme. Several stimuli induce activation of PKDs in cardiomyocytes. Endothelin, growth factors and α-adrenergic agonists activate distinct members of the family of G protein-coupled receptors (GPCR), leading to sarcolemmal phospholipase C activation and subsequent increases in diacylglycerol (DAG) and Ca^2+^. DAG, as part of the canonical PKD1 activation pathway, binds to PKD1 thereby bringing it to the sarcolemma for further activation by PKCs. From there, the activated PKD1 migrates either to the nucleus or to endosomes. Please note that the sarcolemmal activation step of the cytoplasmic PKD1 pool is not displayed. In the nucleus, PKD1 phosphorylates the histone deacetylases (HDAC)5 and HDAC7. HDAC5 phosphorylation results in release and activation of the hypertrophic transcription factor MEF2. HDAC7 phosphorylation leads to binding to and inhibition of FOXO1, thereby depressing CD36 expression and cellular fatty acid uptake. In the cytosol, DAG stimulates PKD1 to migrate to the endosomes to acutely stimulate GLUT4 translocation and glucose uptake. Increased Ca^2+^ stimulates calcineurin-induced upregulation of PKD3 and activation of three additional hypertrophic transcription factors (GATA, NFAT, and NKx). PKD3 also regulates sarcolemmal GLUT1 localization. An increase in physical work (contractile activity) increases mitochondrial formation of ROS as by-product of elevated oxidative phosphorylation flux. DAPK migrates to the endosomes to activate the endosomally resident PKD1 pool to initiate vesicle-mediated GLUT4 translocation. DAPK, as well as Abl/Src may stimulate phosphorylation of myofilament proteins thereby further stimulating contractile activity. mROS production activates the Ser/Thr-kinase DAPK and the Tyr-kinases Abl and Src. For clarity, the effects of the PKD are divided into acute (signaling, metabolism and contraction; *left part of figure*) and chronic (nuclear; *right part of figure*). It is of importance to note that PKD1 and PKD3 integrate cardiac metabolism with contraction and also with remodeling. In case of integration of metabolism with contraction (as occurs upon an acute increase in workload), PKD1 activation ensures that the increase in contractility occurs hand in hand with increased glucose uptake. If the workload transcends from acute into chronic (as occurs upon chronic pressure overload), the PKDs are also needed for integration of metabolism with remodeling. Specifically, chronically elevated GLUT4 translocation and elevated GLUT1 expression together lead to increased glucose uptake and subsequent O-GlcNAcylation of myocellular proteins including the hypertrophic transcription factors. Together, the PKD1/PKD3-mediated direct activation of the transcription factors and the PKD1/PKD3 mediated O-GlcNAcylation and further activation of the transcription factors may lead to full-blown hypertrophic cardiac remodeling.

PKD1 is the most intensively studied member, and is not only activated by DAG, similarly to the classical and novel PKCs, but also directly by the novel PKCs themselves, i.e., via phosphorylation at two serines (human Ser738/Ser742, rodent Ser744/Ser748) within the activation loop, which is situated in the catalytic domain (Figure 1). This PKC-mediated PKD1 phosphorylation is initiated by binding of growth factors, secretory peptides (like endothelin) or α-adrenergic agonists (like phenylephrine) to G protein-coupled receptors (GPCRs), leading to activation of phospholipase-C and formation of DAG and subsequent PKC activation (Figure 2). The latter triggers a conformational change within PKD1 leading to release of auto-inhibition and increased auto-phosphorylation at the extreme C-terminal end (human Ser910, rodent Ser916). This mechanism of PKD activation is now regarded as the canonical PKD signaling pathway. Furthermore, auto-phosphorylation of PKD1 likely reflects activation, although this might not hold for every circumstance (14).

Interestingly, there are subtle differences between PKD1 activation by endothelin and by the α-agonist phenylephrine, which stimuli operate through closely related yet slightly different GPCRs (15). Both stimuli induce PKD1 translocation to the sarcolemma, which brings it in close contact to its upstream kinases to undergo subsequent release of its auto-inhibition. However, endothelin-induced PKD1 association with the sarcolemma is more long-lasting. Additionally, PKD1 translocation to the nucleus is more pronounced in response to phenylephrine compared to endothelin (16).

Another mechanism of PKD1 activation occurs in response to oxidative stress. Increased levels of mitochondrially produced reactive oxygen species (mROS) cause a series of tyrosine phosphorylation of PKD1 extra to the activation loop Ser-phosphorylations of the canonical activation pathway. These tyrosine phosphorylations are mediated by proto-oncogene tyrosine kinases Abl and/or Src, and precede the Ser phosphorylations (for details, see Figure 1). Especially, phosphorylation at human Tyr463 (rodent Tyr469) within the PH domain is needed for full PKD1 activation (17), and initiates docking of PKD1 at the mitochondria (18). Additionally, mROS-induced phospholipase D1 activation and generation of DAG is needed to support binding of PKD1 to mitochondrial membranes. ROS-induced PKD1 activation results in a markedly different pattern of activation of downstream kinases compared to the canonical pathway, and also sarcolemmal PKD1 translocation is not involved (19). mROS-induced PKD1 activation plays an important role in the development of defense mechanisms in tumor cells upon increased oxidative stress (19).

Besides the cytoplasmic pool of PKD1, which translocates to the plasma membrane or nucleus in response to GPCR agonists and oxidative stress there is also a PKD1 population localized to the trans-Golgi—endosomal continuum, as reported for several non-muscle cell lines under non-stimulated conditions (20, 21). Trans-Golgi PKD1 is involved in vesicle transport between endosomes and the plasma membrane. Also in rodent cardiomyocytes there is an endosomal population of PKD1, contributing to of total cellular PKD1 under non-stimulated conditions (22). Using an *in vitro* kinase assay, we found this pool to be activated upon contraction stimulation (22). This activation was accompanied by Ser916 auto-phosphorylation without any trace of Ser744/748 trans-phosphorylation. Hence, contraction-induced PKD1 activation in the heart has a unique signature, most likely via DAPK (as explained in the next section), which is different from GPCR agonist-induced or Abl/Src-mediated PKD1 activation (14).

In greater perspective, PKD activation is cell-type and stimulus-dependent. Moreover, PKD1 resides at different subcellular locations (i.e., cytosol and trans-Golgi/endosomes), and these different PKD1 pools are subject to activation by distinct stimuli (Figure 2).

## PKD1 and regulation of glucose uptake

Contraction is a main physiological stimulus impacting on cardiac metabolism. An increase in contractile activity markedly increases the metabolic demands of the heart for ATP generation which, therefore, results in the increased oxidation of glucose and (long-chain) fatty acids. In non-contracting primary cardiomyocytes *in vitro*, both respective membrane transporters GLUT4 and CD36 are largely present in intracellular membrane compartments (recycling endosomes). Electrically-induced contractions stimulate the translocation of transporters from these intracellular storage compartments (endosomes) to the cell surface within minutes, thereby increasing the uptake of both glucose and fatty acids (4). PKD1, identified as a contraction-activated kinase is involved in contraction-stimulated regulation of cardiac substrate uptake. Studies with PKD1-silenced HL-1 cardiomyocytes and with cardiomyocytes from cardio-specific PKD1 knockout mice have shown that acute contraction-induced GLUT4 translocation and glucose uptake are dependent on PKD1 activation, while contraction-induced CD36 translocation and fatty acid uptake do not need PKD1 (9). Hence, PKD1 activation has the potential to shift the cardiac substrate preference away from fatty acids toward increased glucose utilization (23). This role of PKD1 in GLUT4 translocation resembles the role of PKD1 in Golgi transport and secretion of proteins in non-muscular cells. In both cases, the same subcellular pool of PKD1 may be activated, being the PKD1 pool resident at the Golgi/endosomes. Furthermore, in both cases PKD1 activation initiates budding of vesicles for translocation to the cell surface. In case of Golgi-mediated transport of secretory proteins, PKD1 phosphorylates and activates a lipid kinase, phosphatidylinositol-4 kinase (21), which initiates curvature into the bilayers and also serves as platform for scission proteins involved in vesicle excision (24). Similarly, in the endosomes there may be similar lipid kinases present to support vesicle budding in response to PKD1 activation. Indeed, several other Golgi/endosomally present proteins involved in vesicle budding possess a PKD consensus motif, such as CtBP3/BARS or diacylglycerol kinase (24).

The underlying mechanism by which contraction stimulates PKD1-mediated GLUT4 translocation was pinpointed to ROS levels. This is based on the observation that ROS levels are increased in contracting cardiomyocytes, and that both contraction-induced PKD1 activation and GLUT4 translocation are sensitive to inhibition by ROS scavengers (9). As mentioned in the previous section, contraction-induced PKD1 activation does not require PKD1 Ser744/748 trans-phosphorylation, thereby proposing that contraction induces a novel unique activation mechanism of PKD1. This novel activation mechanism was found to include death-activated protein kinase (DAPK), a kinase mainly known from the cancer field. DAPK induces cellular apoptosis upon several death-inducing signals [for review see (25)]. In line with this, DAPK is proposed to act as a tumor suppressor, as its expression is lost in several human malignancies (26). Just like PKD1, DAPK is activated by ROS, as observed in HEK293T cells in which DAPK undergoes an activating Ser308 de-phosphorylation upon H_2_O_2_ stimulation (27). Additionally, DAPK and PKD1 physically interact with each other upon contraction stimulation of cardiomyocytes, which binding is necessary for PKD1 activation (9). This mechanism closely resembles oxidative stress-mediated PKD1 activation in HEK293T cells, which is dependent on DAPK-Ser308 de-phosphorylation and subsequent DAPK–PKD1 interaction independent of PKCs and PKD1-Ser744/748 trans-phosphorylation (27). The role of DAPK in glucose uptake was assessed in HL-1 cells, in which DAPK was silenced via siRNA transfection. Not only was contraction-induced GLUT4 translocation abolished, also CD36 translocation was unimpaired (9), thereby providing additional support for the existence of a DAPK–PKD axis in selective glucose uptake into the heart. In conclusion, ROS activates DAPK via a yet unidentified phosphatase, which then travels to the endosomes to bind to and activate PKD1, which on its turn activates an endosomal budding kinase to stimulate formation of GLUT4-containing vesicles that translocate to the cell surface (Figure 2).

There is also some evidence that PKD1 activation in the heart upon α-adrenergic stimulation leads to acute increases in glucose uptake. It is known for more than two decades that α-agonists, such as epinephrine (28) and phenylephrine (29), induce both glucose uptake and GLUT4 translocation in cardiomyocytes in an insulin-independent manner. It is presumed that the α-agonists use the canonical activation pathway, including activation of novel PKCs and transphosphorylation of cytoplasmic PKD1 pools at Ser744/748, which then migrate to endosomes to induce GLUT4-vesicle budding.

## PKD1 and regulation of lipid uptake

As stated in the introduction, CD36 is the main cardiac fatty acid transporter, and therefore a major site of regulation of lipid utilization in the heart. Interestingly, contraction-stimulated PKD1 activation does not induce CD36 translocation to the cell surface. This is remarkable because CD36 and GLUT4 are stored at least partly within the same intracellular compartments, i.e., the endosomes (4). At present it is unknown why only GLUT4 (not CD36) is sorted into transport vesicles during PKD1 activation.

At the level of regulation of CD36 expression, PKD1 activation downregulates transcription of the CD36 gene (30). This was shown in endothelial cells exposed to angiogenic growth factors, thereby activating PKD1 via the canonical pathway including PKCs. Subsequently, PKD1 increases phosphorylation and nuclear accumulation of HDAC7, which then associates with the transcription factor FoxO1 and depresses CD36 transcription (30). Whether PKD1 activation also directly decreases expression of CD36 in the heart via this pathway is not known, but seems likely. Specifically, in hypertrophic hearts from rodents that have undergone a transverse aortic constriction, there is an upregulation of PKD1-Ser916 phosphorylation (31) and also a downregulation of CD36 expression (32). Hence, it appears that PKD1 activation (acutely) upregulates cardiac glucose utilization and (chronically) downregulates cardiac fatty acid uptake and utilization.

In contrast, another report has suggested that PKD1 activation leads to increased cardiac lipid utilization. This study reported that the upregulation of lipoprotein lipase (LPL) activity upon experimental induction of type-1 diabetes in rats is PKD1 dependent (33). LPL mediates the hydrolysis of triacylglycerol-rich lipoproteins to fatty acids, thereby contributing to increased fatty acid delivery to the heart. PKD1 activation in these diabetic animals was due to phosphorylation of heat shock protein-25 (HSP25) by unknown mechanisms, thereby dissociating from and de-inhibiting the novel PKC isoform PKCδ, subsequently resulting in PKD1-Ser744/748 transphosphorylation. The latter was associated with secretion of LPL, in line with the role of PKD1 in Golgi transport and vesicle budding (21). However, it has not been assessed whether PKD1-mediated LPL secretion leads to increased cardiac fatty acid utilization. Such PKD1-mediated LPL upregulation would contrast the PKD1 mediated downregulation of CD36. The apparent paradox may be solved by the possibility that these opposing actions of PKD1 may not operate under the same circumstances. For instance, LPL upregulation may be confined to specific animal models of type-1 diabetes, while CD36 downregulation may be a characteristic feature of the pressure-overloaded heart.

## PKD1 and regulation of myofilament contractility

In a yeast-two hybrid screen of a human cardiac library using a catalytically inactive mutant of the catalytic domain of PKD1 as interaction partner, several myofilament proteins were identified as potential downstream targets, including troponin-I (TnI), telethonin, and cardiac myosin-binding protein-C (cMyBP-C) (34). This implemented PKD1 as potential regulator of myocyte contractility. Some of these phosphorylation sites overlap with protein kinase-A (PKA) phosphorylation sites, suggesting that PKD activation, just like β-adrenergic stimulation, leads to increased cross-bridge cycle kinetics and increased force development [see review 13). The phosphorylation of TnI by PKD1 at Ser22/23 has been shown to alter myofilament Ca^2+^ sensitivity (35). This phosphorylation appears to be a monophosphorylation, and not a bisphosphorylation as mediated by PKA (36). The phosphorylation of cMyBP-C by PKD1 at Ser315 does not alter myofilament Ca^2+^ sensitivity but increases maximal tension of contraction. Thus, the combined actions of PKD1 on distinct myofilament proteins fine-tune the increase in force development during times of increased cardiac contractile activity.

It is not yet known which of the PKD activation routes are involved in contraction-induced phosphorylation of myofilament proteins. Most likely it is a ROS-induced signaling pathway. It could be the ROS-activated Abl/Src pathway leading to PKD1-Tyr95/463 phosphorylation, although activation of PKD1 in an Abl/Src dependent manner has not yet been investigated in the heart. Alternatively, DAPK could be involved in these contractile protein phosphorylations. In relation to GLUT4 translocation, DAPK would selectively activate the endosomal PKD1 population to induce endosomal vesicle budding. For contractile protein phosphorylation, DAPK would additionally need to activate cytoplasmic pools of PKD1 for subsequent migration to the contractile apparatus. This topic requires further studies.

## PKD1 and cardiac hypertrophy

The canonical PKD1 activation pathway has been implicated in the pathological remodeling events in the heart that occur in response to hypertension. During hypertension, a number of GPCR agonists is elevated such as endothelin and several catecholamines, which activate PKD1 in a PKC-dependent manner and via phosphorylation at Ser744/748, i.e., the canonical PKD1 pathway. A key substrate of PKD1 in hypertension-induced cardiac remodeling is histone deacetylase-5 (HDAC5), a member of the HDAC family negatively regulating the acetylation status of nucleosomal histones, and thereby repressing transcription. Moreover, HDAC5 is a negative regulator of cardiac remodeling via binding to the myocyte enhancer factor-2 (MEF2). MEF2 transcription factors contain a MEF2-specific domain and an adjacent dimerization domain (named MADS box) (37), allowing these transcription factors to bind as dimer to specific A/T-rich sequences in enhancer regions of a number of fetal genes. Increased transcription of these genes in the adult heart results in a hypertrophic phenotype. PKD1 phosphorylation of HDAC5 induces HDAC5 binding to the adaptor protein 14-3-3 and subsequent disassociation from MEF2. This de-repression of MEF2 then switches on the fetal gene program. The precise mechanisms controlling nuclear import of Ser744/748-transphosphorylated PKD are incompletely understood, but do include another adaptor protein, AKAP13, from the A-kinase-anchoring proteins (AKAPs) acting as scaffolds for signaling proteins. AKAP13 is predominantly expressed in the heart and mediates PKD1 phosphorylation by bringing PKD1 and PKC in close proximity (38). AKAP13 is also involved in subsequent transport of PKD1 into the nucleus. Ablation of the PKD1 binding domain of AKAP13 leads to a decreased hypertrophic response upon hypertrophic stimulation. Interestingly AKAP13 expression is increased upon the onset of cardiac hypertrophy (38).

Recently, another mechanism via which PKD1 may contribute to cardiac hypertrophy, has been revealed by using the transverse aortic constriction (TAC)-induced cardiac hypertrophy mouse model (39). This novel mechanism includes an inhibitory action of PKD1 on autophagy via upregulation of the Akt/mTOR pathway.

As expected, PKD1 activation is indeed observed in the hypertrophic heart, as in mice that have undergone TAC surgery, there is a marked increase in PKD1 phosphorylation (31, 39). Powerful genetic evidence for a crucial role of PKD1 in cardiac hypertrophy comes from studies with mice with cardio-specific overexpression of a constitutively active PKD1 mutant. These mice develop left ventricular chamber dilatation, myocyte hypertrophy, and wall thinning already early in life together with a severe impairment of fractional shortening (40).

## PKD2 and PKD3 and their roles in cardiac energy metabolism and pathophysiology

PKD1 is by far the most abundant isoform in the heart, but the other two isoforms are also present and appear to fulfill several non-redundant roles in cardiac metabolism and pathophysiology (41). PKD2 most closely resembles PKD1 as it also contains the C-terminal auto-phosphorylation site, i.e., at Ser876, while also the flanking amino acid sequences are conserved. In contrast, the N-terminal sequences are highly variable between PKD1 and PKD2 (and also PKD3), which might explain the agonist-specific regulation of each isoform. For instance, PKD2 is not activated by the α-agonist norepinephrine (41). Moreover, PKD2, but not PKD1, auto-phosphorylation is upregulated in hearts of *db/db* mice, a mouse model of diabetic cardiomyopathy (42). Remarkably, increased PKD2 phosphorylation was not accompanied by changes in phosphorylation of HDAC5, the classical PKD1 substrate so that the observed hypertrophy is not due to activation of the MEF2-hypertrophic program. Treatment of *db/db* mice with the pan-PKD inhibitor CID755673 decreased the cardiac hypertrophy and restored cardiac function, but strangely did not affect HDAC5 phosphorylation (42). Unfortunately, this study did not describe cardiac function in *db/db* mice in which the PKD2 gene was ablated, so that off-target actions of this drug cannot be excluded. In contrast to *db/db* mice, increased PKD1 and PKD2 auto-phosphorylation was not detected in mice fed a Western (45 en% fat) diet, another model of diabetic cardiomyopathy (23). Hence, the role of PKD2 in the development of cardiac hypertrophy in *db/db* mice awaits further investigations.

PKD3 is more different from PKD1 than PKD2, as it is missing the C-terminal autophosphorylation domain, and therefore displays a slightly smaller molecular mass (90 vs. 115 kDa for PKD1 and 105 kDa for PKD2). Remarkably just like PKD1, PKD3 is also involved in regulation of glucose uptake, as established in skeletal muscle cell lines (42), but not yet confirmed in the heart. However, PKD3's involvement is different: in contrast to PKD1, PKD3 is not regulating stimulus-induced GLUT4 translocation and glucose uptake, but regulates the subcellular localization of GLUT1 and basal glucose uptake (43). Also remarkably, PKD3, just like PKD1, is involved in mediating the morphological and functional changes as seen during the development of cardiac hypertrophy. The transcription factor MEF2, which is upregulated upon PKD1 activation, is not the only transcription factor mediating the full pleiotropy of changes displayed by the hypertrophic heart. Also other transcription factors contribute to these changes, among which nuclear factor of activated T-cells (NFATc4), NK family of transcription factor 2.5 (Nkx2.5) and GATA4 [for review see (44)]. In isoproterenol-induced hypertrophy, PKD3 is substantially upregulated, and this upregulation is required for increased expression of the latter three transcription factors, whereas PKD3 does not regulate MEF2 (45). The upregulation of PKD3 expression in isoproterenol-treated neonatal cardiomyocytes is due to activation of the Ca^2+^-sensitive phosphatase calcineurin upon increased Ca^2+^ levels. Calcineurin then stimulates the nuclear import of the transcription factors NFATc1 and NFATc3, followed by upregulation of PKD3 expression (45). It remains to be investigated if this PKD3 involvement, as seen in neonatal cardiomyocytes, can be reproduced in the adult heart.

In conclusion, the role of PKD2 in cardiac metabolism and remodeling is still unclear, whereas PKD3, in conjunction with PKD1, appears to regulate both glucose uptake and cardiac growth in a non-redundant manner.

## Integration of roles of PKD1 and family members in cardiac energy metabolism and pathophysiology

From the evidence collected over the last two decades there now is consensus that the PKD family—especially PKD1—plays a key role in cardiac metabolism, morphology and function. First of all, upon an acute increase in cardiac workload as occurs at the onset of physical work (e.g., exercise), PKD1 becomes rapidly activated, leading to the combined phosphorylation of contractile proteins and of a protein component of the vesicle budding machinery in the recycling endosomes. This then leads to combined increases in contractile force and in GLUT4-mediated glucose uptake. Hence, PKD1 synchronizes the increased mechanical performance of the heart with the increased energetic demands in order to appropriately react to an acute increase in physical work.

In situations that PKD1 is chronically activated, such as during chronic stress or increased blood pressure, or a combination thereof, it activates in combination with PKD3 a set of transcription factors with established involvement in hypertrophic growth of the heart. The activation of MEF2 is not the only action of PKD1 involved in cardiac hypertrophy. As mentioned in sections “PKD1 and Regulation of Glucose Uptake” and “PKD2 and PKD3 and Their Roles in cardiac Energy Metabolism and Pathophysiology,” PKD1 and PKD3 activation leads to increased glucose uptake. Hence, upon chronical stimulation of GLUT4 translocation and GLUT1 expression by PKD1 and PKD3, respectively, glucose uptake markedly increases and feeds forward to increased glycolytic rates, increased accumulation of glucose metabolites and of glucose-mediated post-translational modifications of proteins (glycosylation and O-GlcNAcylation). Especially protein O-GlcNAcylation is currently regarded as a sensitive indicator of glucose fluxes (46), and is observed to be increased in the hypertrophic heart (47, 48). Among the O-GlcNAcylated proteins are transcription factors, as observed during increased glucose fluxes in cancer cells (49). Possibly, the PKD1/PKD3-activated hypertrophic transcription factors may also undergo increased O-GlcNAcylation, thereby installing a vicious cycle of PKD-mediated actions in the heart on its way to hypertrophy. Specifically, the transcription factor O-GlcNAcylations following increased PKD1/PKD3-mediated myocellular glucose uptake may reinforce the direct activation of these transcription factors by PKD1/PKD3 resulting from PKD1/PKD3 translocation to the nucleus (Figure 2).

On top of this, PKD1 activation decreases CD36 expression in a FOXO1-dependent manner (see section “PKD1 and Regulation of Lipid Uptake”), which further may contribute to the development of cardiac hypertrophy. The consequently decreased fatty acid uptake rates will lead to de-activation of lipid-induced transcription factors such as members of the family of peroxisome proliferator-activated receptors (PPARs) (50). Because, especially PPARα activation has been shown to be cardio-protective in the hypertrophic heart (51), de-activation of this transcription factor may further worsen the metabolic and energetic state of the hypertrophic heart.

Considering these various PKD actions together in a chronic setting, it becomes increasingly evident that the PKD family is a major novel player in the development of cardiac hypertrophy. This notion is boosted by the observation that PKD1 overexpression in the heart is sufficient by itself for the development of a severe form of cardiac hypertrophy. For future research, it may be of interest to investigate which of these PKD1-stimulated pathways is ultimately responsible for this hypertrophy. It is also conceivable that either pathway by itself is not sufficient, and it is the combination of all these PKD1/PKD3-stimulated pathways that drives maladaptive hypertrophic growth.

Based on the foregoing, the hypertrophic heart displaying a substrate switch toward increased glucose utilization might benefit from drugs that specifically inhibit PKD1, especially in advanced stages of cardiac hypertrophy (pathological remodeling). This would counteract the hypertrophic programming and the chronic GLUT4 translocation, thereby giving the heart the ability to switch back to lowered glucose and increased fatty acid utilization. On the other hand, pharmacological drugs that would specifically induce PKD1 activation might be beneficial for the treatment of the condition of diabetic cardiomyopathy. This is especially relevant since heart failure is the leading cause of death in type-2 diabetic patients. The type-2 diabetic heart is characterized by massive lipid accumulation which over time induces insulin resistance, decreased glucose utilization and ultimately loss of contractile force [for review see 52). PKD1 activation may reverse the state of decreased glucose utilization, by forcing GLUT4 translocation independently of the defective insulin signaling pathway. This would be expected to also normalize fatty acid uptake via Randle cycle-mediated inhibition (53). Examples in rodent studies that this strategy might be successful include the observation that cardio-specific PKD1 overexpressor mice are resistant to develop insulin resistance upon a Western (45 en% fat) diet (23). Furthermore, PKD1 activation would also explain the observation that TAC surgery restored cardiac substrate uptake in *db/db* mice, a mouse model of type-2 diabetes displaying cardiac lipid accumulation and decreased glucose uptake (31). In this model the chronic PKD1 activation following pressure overload would restore glucose uptake to subsequently lead to normalization of the elevated fatty acid fluxes.

In conclusion, PKD is expected to be an attractive target to treat the failing heart whereby it would be dependent on the specific metabolic state of the heart whether to use PKD inhibiting drugs vs. PKD activating drugs. As a result, PKD offers a potential target for personalized medicine.

## Author contributions

OS and JL wrote the first draft of the manuscript. AS, JG, and MN reviewed and edited the manuscript. Final editing was done by MN. All authors read and approved the submitted version.

### Conflict of interest statement

The authors declare that the research was conducted in the absence of any commercial or financial relationships that could be construed as a potential conflict of interest.
